# Food-induced anaphylaxis: our experience

**DOI:** 10.1186/2045-7022-1-S1-P56

**Published:** 2011-08-12

**Authors:** Camilla Di Paolo, Massimo Cinquini, Stefano Minetti, Elena Schlanser, Cinzia Tosoni, Fabio Lodi-Rizzini

**Affiliations:** 1 Medicine - University of Brescia, Allergy Unit, Brescia, Italy; 2 Spedali Civili, Allergy Unit, Brescia, Italy

## Background

food-induced anaphylaxis is the most common single cause of anaphylaxis treated in Emergency Department.

## Method

25 outpatients (15 females, 10 males; mean age 39 years, range 15-68) who experienced previous anaphylactic reactions, following the exposure to a likely food allergen, were evaluated. The diagnosis of anaphylaxis was performed according to the 2005 criteria. Detailed history and physical examination of patients, skin prick test (SPT) with food commercial extracts and with aeroallergens and panallergens LTP and profilin (Alk), prick-to-prick with fresh foods, and serum specific IgE detection (CAP, Phadia) were performed in order to confirm the causative role of the suspected food. Oral food-challenges with positive tested foods were not performed, because of previous severe anaphylaxis. Only in one patient a single-blind placebo-controlled food-challenge (with tomato, positive to SPT but negative to history) was performed, resulting negative.

## Result

briefly, according to Literature, the main involved foods were nuts, crustaceans and mussels, umbelliferae, but also other foods like buckwheat or rabbit meat or acetic acid were sometimes involved.

## Conclusion

our data confirm the importance of performing prick to prick with fresh allergen sources, as they increase CAP positive predicted value and enable the clinician to test unusual allergens. Commercial extracts of the panallergens profilin and LTP were useful tools both for the diagnosis of food allergy and for future dietary management. Although epinephrine is the treatment of choice in anaphylaxis, and most patients fulfilled at least 3 criteria of anaphylaxis, the drug was administered only in five cases. Fortunately, no patient underwent biphasic reactions, complications, or death.

